# Acromioclavicular Joint Separation: Repair Through Suture Anchors for Coracoclavicular Ligament and Nonabsorbable Suture Fixation for Acromioclavicular Joint

**DOI:** 10.1111/os.12771

**Published:** 2020-09-06

**Authors:** Tao Liu, Fei‐long Bao, Tao Jiang, Guang‐wei Ji, Jian‐min Li, Jörg Jerosch

**Affiliations:** ^1^ Department of Orthopaedic Surgery Qilu Hospital (Qingdao), Cheeloo College of Medicine, Shandong University Qingdao China; ^2^ Department of Orthopaedic Surgery Cheeloo College of Medicine, Shandong University Jinan China; ^3^ Clinic for Orthopaedics and Orthopaedic Surgery Johanna‐Etienne Hospital Neuss Germany

**Keywords:** Acromioclavicular dislocation, Coracoacromial ligaments, Heavy sutures augmentation, Horizontal stability, Suture anchors

## Abstract

**Objective:**

To evaluate the clinical and radiographic outcomes of patients undergoing coracoclavicular (CC) ligament repair by two suture anchors and acromioclavicular (AC) joint (ACJ) fixation using heavy nonabsorbable sutures for the treatment of types III–V ACJ injuries with a minimum of 1‐year follow‐up.

**Methods:**

The clinical and radiographic outcomes of 36 consecutive patients (26 men and 10 women) who underwent anatomic reduction for acute ACJ dislocation using two suture anchors for CC ligament reconstruction and two strands of non‐absorbable stitches for ACJ fixation between December 2013 and December 2018 were reviewed. Two 3.5 mm suture anchors with double‐loaded sutures were separately inserted into the anterolateral and posteromedial portions of the coracoid process. The suture strands were passed through the hole created in the clavicle using 2.0 mm drill and tied over the clavicle. Additional ACJ augmentation using two strands of non‐absorbable heavy sutures was performed in all patients. At 3, 6, and 12 months and last follow‐up visit, the scores on the visual analog scale (VAS), the American Shoulder and Elbow Surgeons (ASES) score, Constant–Murley score, and simple shoulder test (SST) questionnaires were used to provide a final evaluation of shoulder function. Comparison between baseline and treatment results was performed. Radiographic analysis included vertical displacement and horizontal shift.

**Results:**

A total of 29 patients (20 men and nine women) were included in the study. A total of seven, six, and 16 patients had Rockwood type III, type IV, and type V ACJ dislocations, respectively. The mean patient age was 42.8 ± 13.5 years, with a mean follow‐up of 28 months (range, 12–56 months). At the 12‐month follow‐up, the mean ASES score was 92.1 ± 3.5, with a mean pain score of 0.5 ± 0.7 on the VAS and mean Constant–Murley score of 93.0 ± 2.4. The new number of positive answers on the SST was 11.5 ± 0.6. Compared with the baseline, the clinical results improved significantly (*P* < 0.05). No significant difference could be found between the 6‐ and 12‐month follow‐up evaluations (*P* > 0.05). Radiographs showed two partial loss of reduction, whereas no horizontal displacement was found in all patients. One patient developed a superficial wound infection 3 weeks postoperation. The wound healed after routine wound care. No neurovascular complications were recorded.

**Conclusions:**

CC ligament reconstruction using two suture anchors and ACJ augmentation using two strands of non‐absorbable heavy sutures on high‐grade AC dislocation is a reliable technique for restoring stability to the ACJ and can obtain good to excellent clinical results.

## Graphical abstract

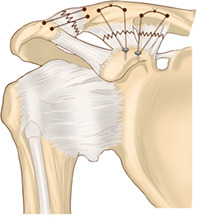

We describe a new technique of CC ligament reconstruction using two suture anchors and ACJ augmentation using two strands of non‐absorbable heavy sutures to restore both vertical and horizontal stability for high‐grade acromioclavicular joint dislocation.

## Introduction

Acromioclavicular (AC) joint (ACJ) dislocations are common injuries and compose a sizeable portion of shoulder injuries^1^. These conditions account for 9% to 12% of shoulder girdle injuries^2^ and are more frequent in young adults and athletes, often resulting from a direct fall onto the superior aspect of the shoulder when the arm is adducted^3^, and five times more common in men than in women. Although the incidence of high‐grade ACJ injuries requiring surgery is low, indications for the conservative versus surgical treatment of type III and V injuries produce controversy^1^, ^3^, ^4^, ^5^, ^6^. This disagreement has encouraged the development of multiple surgical techniques and may reflect a general dissatisfaction with treatment options and outcomes^3^, ^4^, ^6^, ^7^, ^8^.

Numerous surgical repairs or reconstruction techniques have been published. More than 150 techniques for surgical treatment of AC injuries have been described^4^. These techniques generally fall under several categories: AC fixation, coracoclavicular (CC) fixation, or ligament reconstruction. Regardless of technique used, the reduction needs to be maintained long enough for the biological healing process to occur in acute settings^9^. Fixation with one or two suture buttons as an acute repair technique has high biomechanical stability^10^. This technique is optimal for the repair of acutely torn ligaments, providing stabilization to allow native ligaments to heal^11^. The advantage of this procedure is that autograft or allograft or hardware removal is unnecessary^12^, ^13^. High complication rates ranging from 20% to 44% potentially limit its promotion and application. Another concern is the adverse clinical results by residual horizontal instability after CC ligament repair^14^, ^15^.

**Fig. 1:**
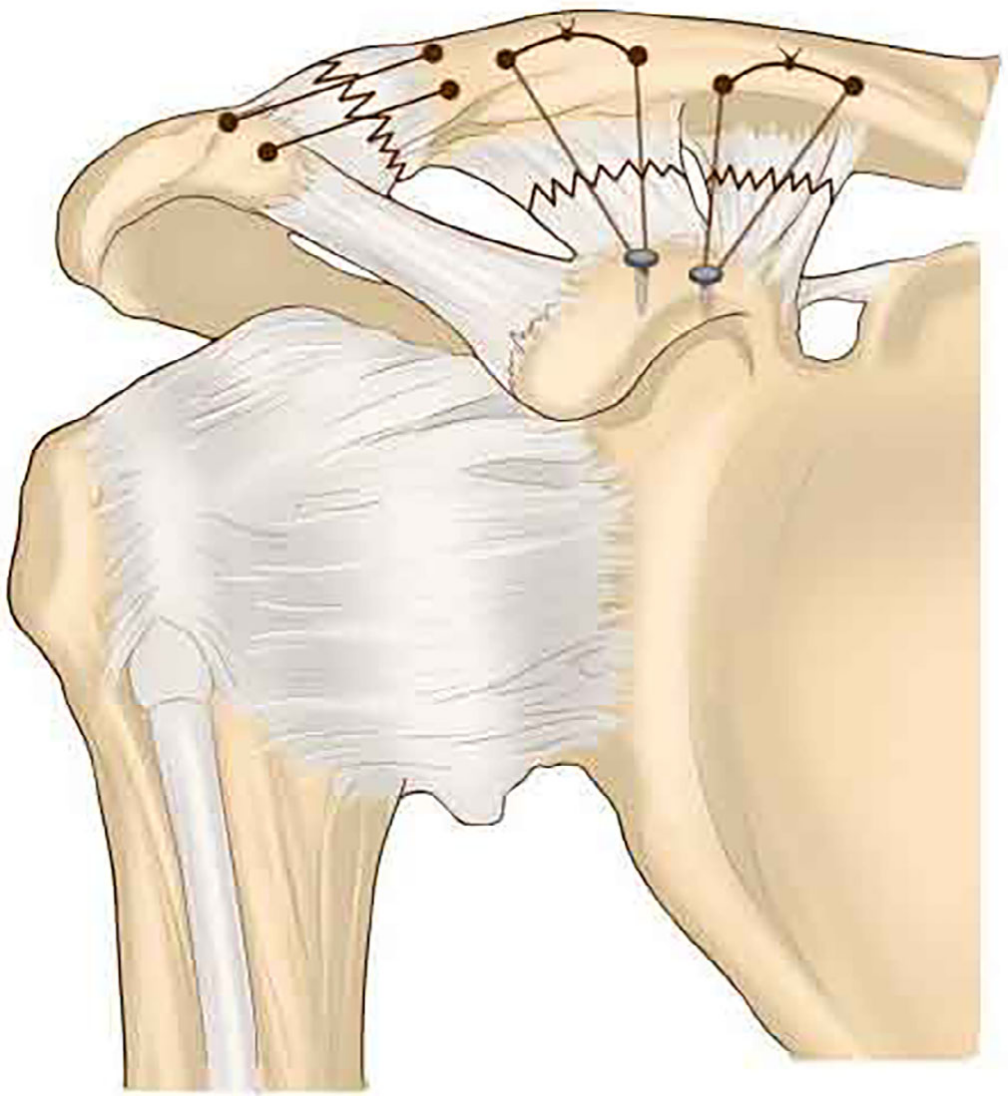
Schematic of four number‐2 Ethibond sutures from two suture anchors were tied over the top of the clavicle for repair CC ligament, and two strands of number‐2 Ethibond sutures crossing the ACJ were tightened for augmentation and restoring horizontal stability.

Given the disadvantages of previously reported procedures, we advocate a new method to address vertical and horizontal stabilities simultaneously. A small diameter tunnel was used to reduce the risk of fractures (Fig. 1). Review of English literature revealed no similar clinical reports. This study aimed to: (i) review the pros and cons of current treatment modalities for ACJ dislocation; (ii) introduce our operative technique in detail, reconstructing both CC ligamnet and AC augmentation; and (iii) report and analyze the radiographic and functional results of patients treated with our methods.

## Materials and Methods

From December 2013 to December 2018, 77 patients with Rockwood^16^ type IV, type V, and unstable type IIIB^17^ AC dislocations were treated surgically in our department. A total of 41 patients received hook plate fixation and were excluded from this study. The inclusion criteria were: (i) all type IV and V dislocations and unstable type IIIB dislocations; (ii) acute (<3 weeks) injuries; (iii) two suture anchors for CC repair and nonabsorbable heavy stitches for AC augmentation; (iv) follow‐up of at least 12 months. The exclusion criteria were: (i) hook plate fixation; (ii) concomitant coracoid fractures; (iii) chronic separations.

**Fig. 2:**
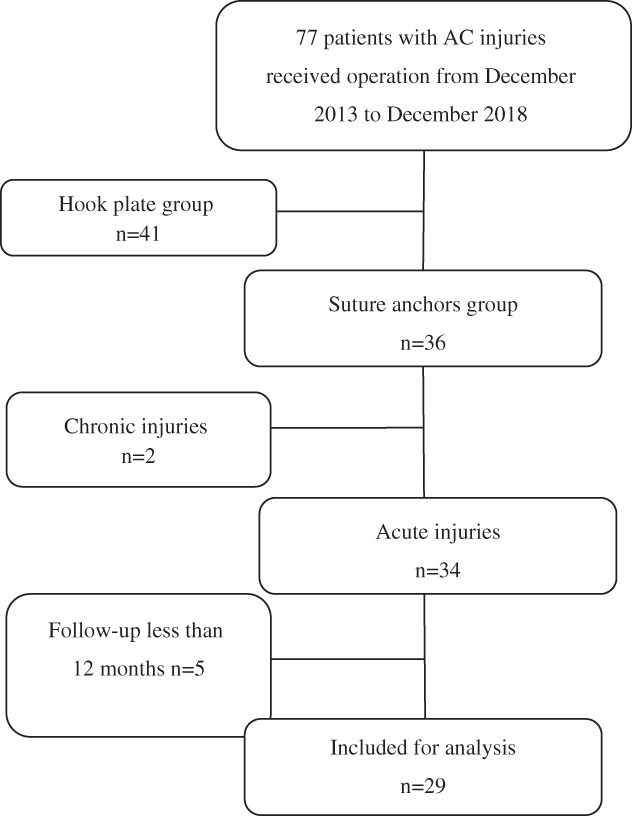
Flow diagram demonstrating the patients included for study analysis.

Of the initial 36 patients who underwent CC ligament and ACJ repair, seven were excluded: two chronic injury patients and five patients who were lost to follow‐up before the 12‐month follow‐up visit. Finally, 29 cases were included in this study (Fig. 2). Table 1 shows the clinical data of patients. A total of seven patients were injured in traffic accidents and 22 by falling during sports. All the 29 patients were prospectively assessed clinically and radiographically preoperatively and at 3, 6, and 12 months postoperatively. Clinical and radiographic data from the 12‐month follow‐up were statistically compared with the baseline. All operations were performed by the same experienced surgeon. The work was approved by the ethical committees in our institution, and patients gave their informed consent.

**TABLE 1:** Patient demographics (N = 29)

Demographics	Data
Sex(N，%)	
Male	20 (69.0)
Female	9 (31.0)
Age, mean ± SD, years	42.4 ± 12.8
Follow‐up, mean ± SD, months	28 ± 10.2
Injury site(N，%)	
Left	16 (55.2)
Right	13 (44.8)
Injury cause(N，%)	
Traffic accident	7 (24.1)
Falling over	22 (75.9)
Fracture type(N)	
III	7
IV	6
V	16
Operation time, mean ± SD, min	77.3 ± 14.1
Bleeding, mean ± SD, mL	67.1 ± 18.6

SD, standard deviation.

## Operative Technique

### *Anesthesia and Position*

The procedure was performed with the patient in beach chair position and under brachial plexus block or general anesthesia.

### *Approach and Exposure*

An incision was made starting at the posterior edge of the clavicle, 2 cm medial to the ACJ and extending inferiorly toward the coracoid process along the Langer line. Dissection was performed to the deltotrapezial fascia with electrocautery. The fascia was elevated off the clavicle by creating full‐thickness flaps. The intra‐articular disc was removed, all soft tissues preventing proper joint reduction were resected, and a trial reduction was performed^8^, ^18^. Effort was made not to excise nor damage the distal clavicle.

### *Vertical Stability Repair*

**Fig. 3:**
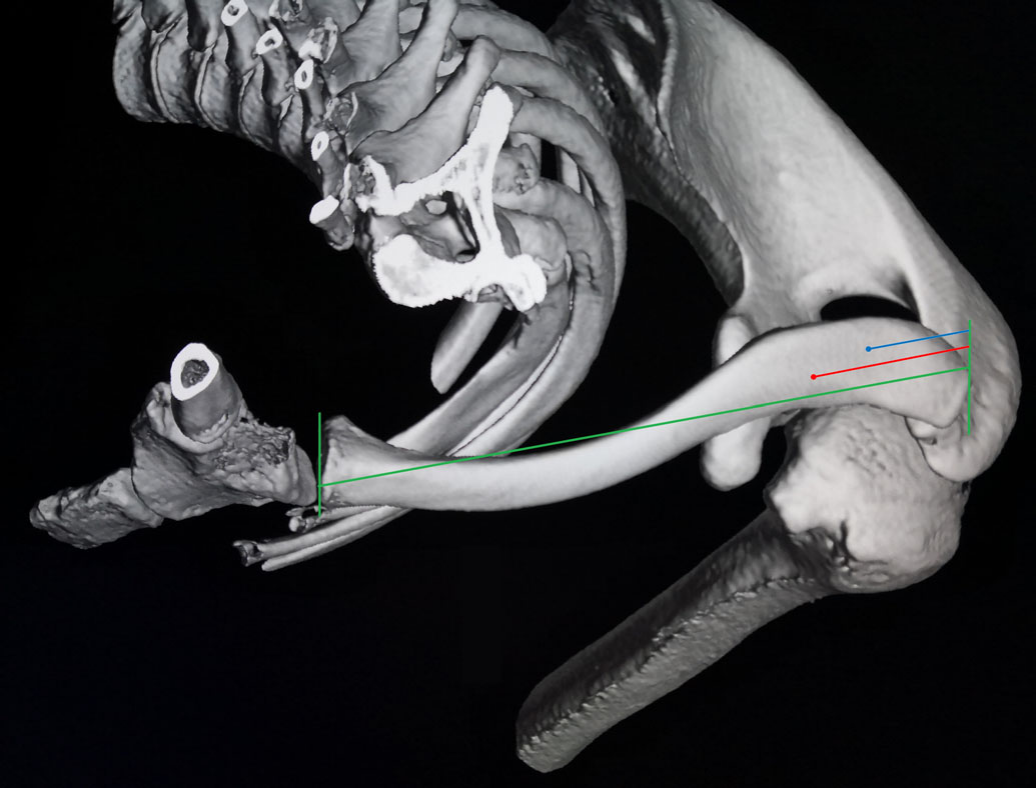
Superior view of 3‐D‐CT reconstruction of a left shoulder showing an example of the planned tunnel location of the conoid (red) and trapezoid (blue) limbs based on anatomic ratios.

**Fig. 4:**
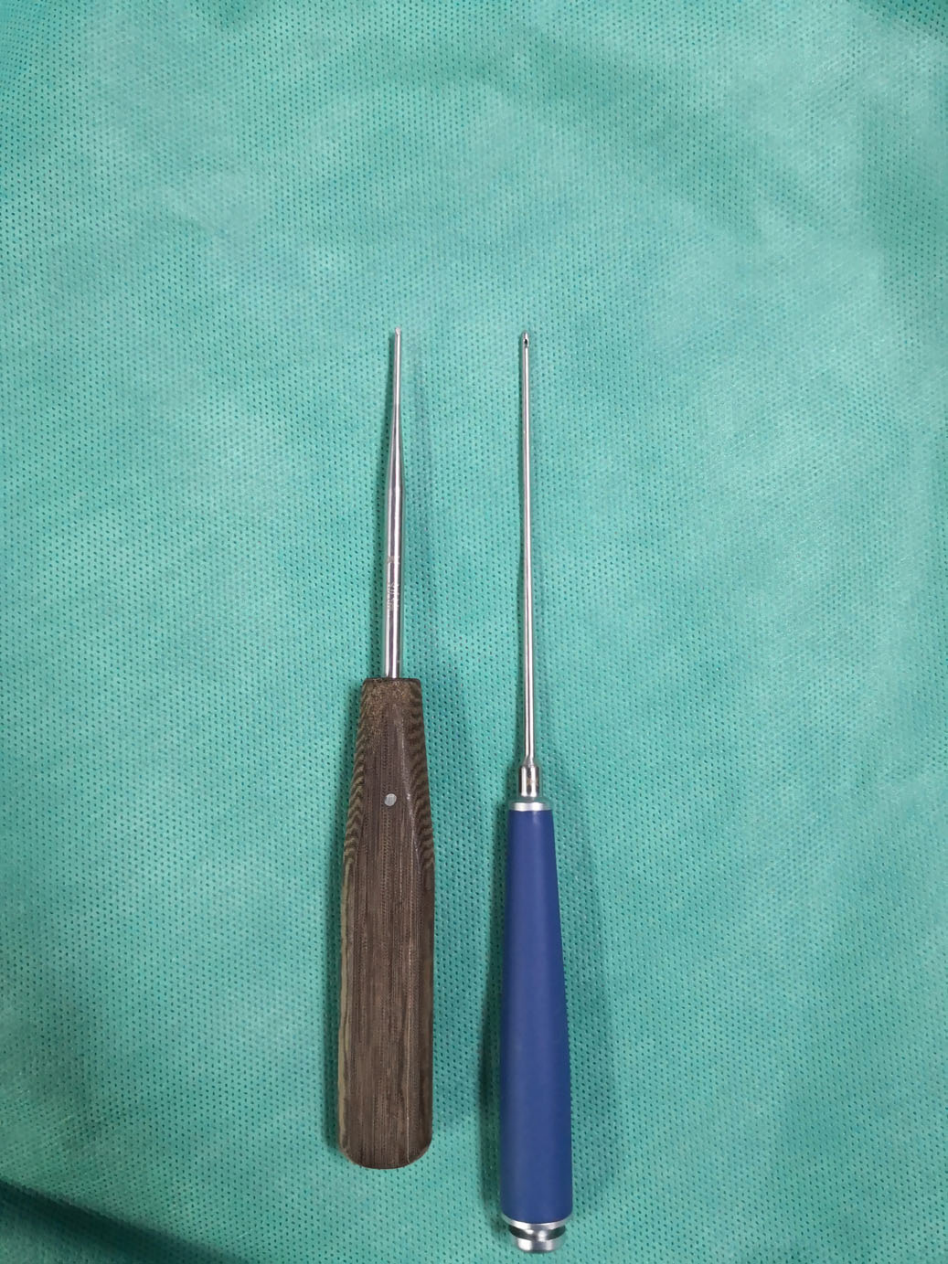
Self‐made special passer (right) and awl (left).

Through dissection, the base of the coracoid process was exposed. Two 3.5 or 5.0 mm (for stronger patients) suture anchors (Twinfix, Smith & Nephew, Memphis, Tennessee, US) with double‐loaded sutures were separately inserted into the anterolateral and posteromedial portions of the coracoid process and matched to the conoid and trapezoid ligament anatomic insertion^19^. The clavicle was preoperatively templated to place the conoid tunnel at 20% to 25% of the clavicular length from the distal clavicle, and the trapezoid tunnel was placed 1.5 cm to 2 cm lateral to this position (near the anatomic insertion at 17% of clavicular length) (Fig. 3)^20^. Two holes, at least 1 cm apart, were created in the clavicle with a 2.0 mm drill for conoid and trapezoid ligament insertion separately. A special passer (Fig. 4B) was used to assist in passing the loaded sutures of anchors quickly. The sutures were left for later tightening.

### *Horizontal Stability Reconstruction*

**Fig. 5:**
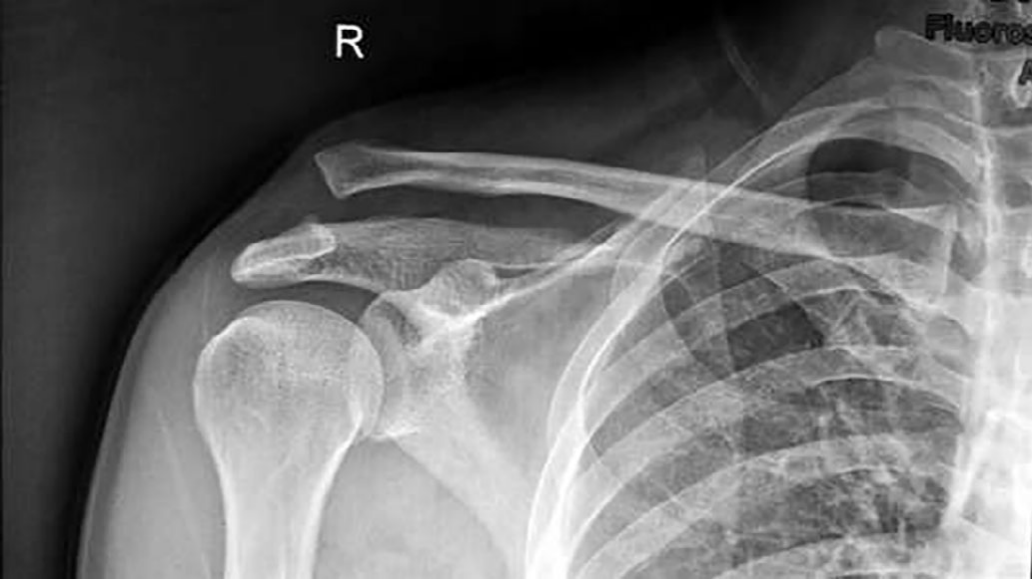
Male patient, 42 years old, Rockwood type V right AC dislocation.

**Fig. 6:**
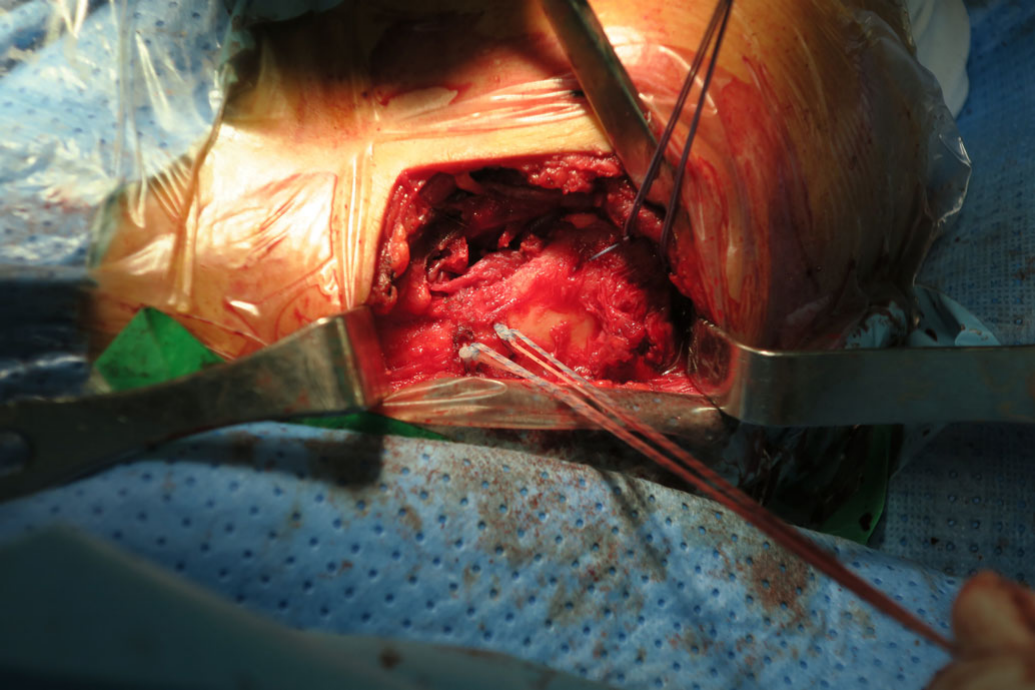
Intraoperative, superior view, two suture anchors with four number‐2 Ethibond sutures for CC ligament and two number‐2 Ethibond sutures for ACJ.

**Fig. 7:**
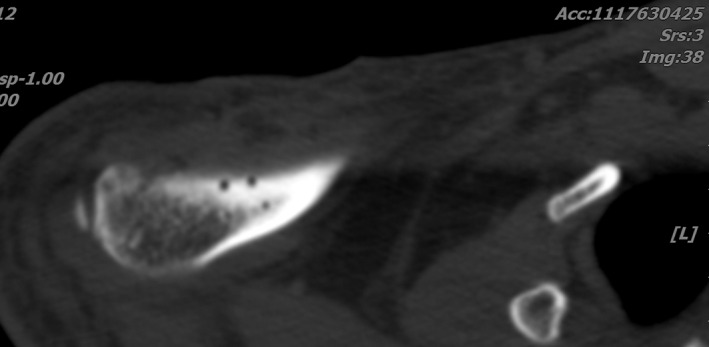
Postoperative CT‐scan shows four holes for CC ligament repair.

**Fig. 8:**
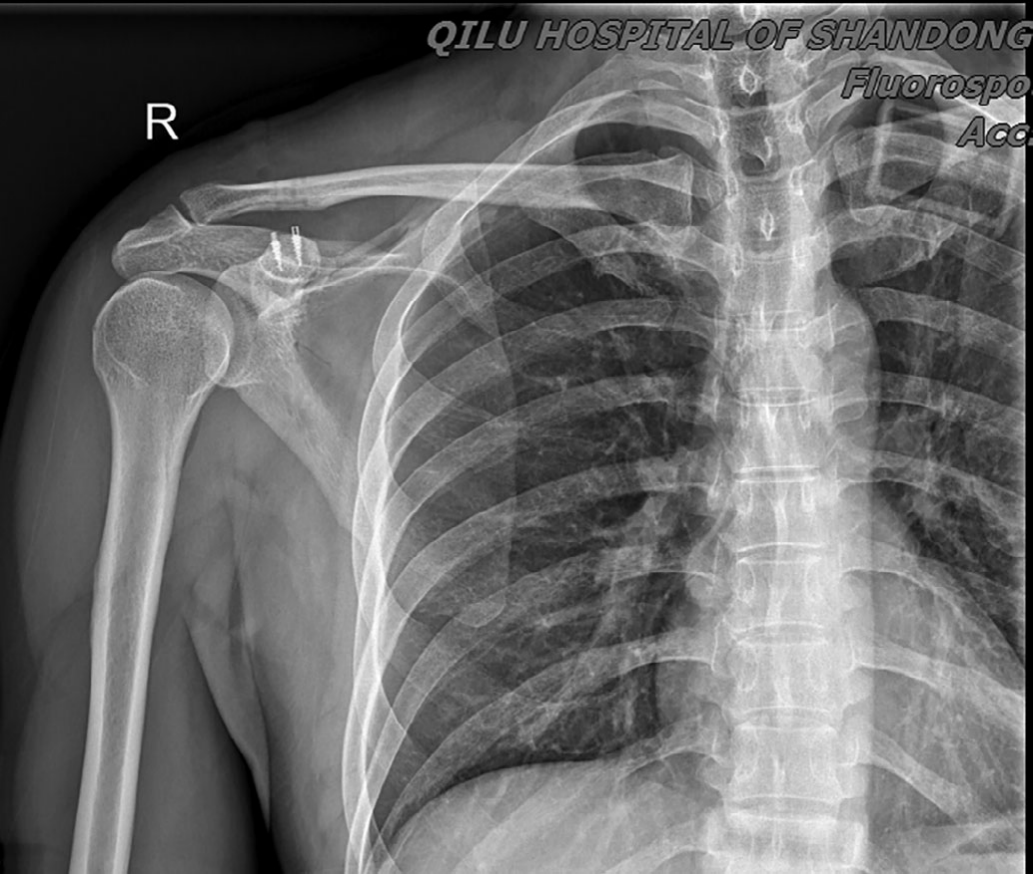
X‐Ray image at 12‐Month follow‐up demonstrating the good position of ACJ.

**Fig. 9:**
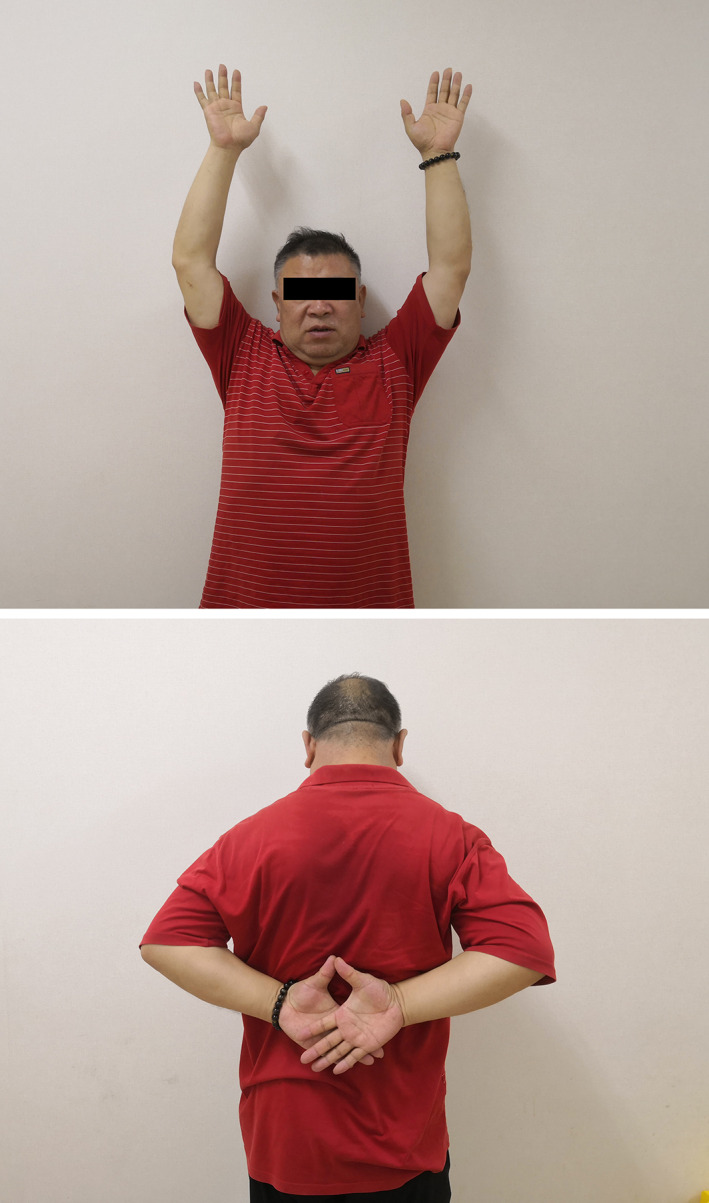
The patient obtained full range of motion of injury shoulder and no pain.

To horizontally stabilize the ACJ, we created two tunnels by using a special 2 mm‐diameter awl (Fig. 4A). The tunnel started from the acromion, passed through the ACJ, and obliquely exited the superior surface of distal clavicle 1 cm from ACJ. Two number‐2 Ethibond sutures (Ethicon, Somerville, New Jersey, US) were then pulled through the holes of the acromion and distal clavicle separately. The dislocated ACJ was reduced under direct vision with shoulder abduction by manually pressing down the distal end of the clavicle. After reduction of the ACJ, the sutures on the superior surface of clavicle for CC ligament repair were tightened and tied, followed by tightening of the sutures on the distal clavicle for ACJ augmentation. After repairing the AC ligament and capsule, the stability was then assessed by passively moving the shoulder. The deltotrapezial fascia was carefully repaired, and a routine wound closure was performed (a typical case is shown in Figs 5, 6, 7, 8, 9).

### *Postoperative Management*

Postoperative rehabilitation included wearing a strict sling for 6 weeks. Passive shoulder motion was begun at 3 weeks, and exercises against resistance were subsequently added at 6 to 8 weeks postoperatively. Motion was gradually increased after cessation of sling wear with a goal of full motion at 3 months. Strengthening started at this point, and patients were allowed to return to contact sports at 6 months^5^, ^21^.

### *Follow‐up Analysis*

Follow‐up ranged from 12 to 56 months, with an average of 28 months. At the 3‐, 6‐, and 12‐month and the latest follow‐up, radiographic analysis and visual analog scale (VAS), American Shoulder and Elbow Surgeons (ASES) score, Constant–Murley score, and simple shoulder test (SST) questionnaires were utilized for the final evaluation of shoulder function.

### *Radiological Assessment*

Anteroposterior radiographs of both ACJs were produced for each patient. Axillary radiographs were obtained for the injured side only. Maintenance of vertical reduction of the ACJ was defined as follows: (i) a maintained reduction, that is, no side‐to‐side difference on the anteroposterior radiographs; (ii) a partial loss of reduction, that is, a side‐to‐side difference of less than the width of the clavicle; (iii) complete loss of reduction, that is, evidence of a side‐to‐side difference in excess of the clavicle width^21^. Horizontal stability was assessed by axillary view and three‐dimensional computed tomography (3‐D‐CT). The anterior tip of acromion and anterolateral edge of the distal clavicle were in line or at displacement less than 2 mm with the ACJ in anatomical position, indicating no subluxation nor dislocation in terms of horizontal instability. Anterior–posterior displacement exceeding 2 mm was defined as horizontal instability^22^, ^23^.

### *Visual Analog Scale (VAS)*

The Visual Analog Scale (VAS) is used in epidemiological and clinical studies to evaluate subjective phenomena, such as the extent of pain, fatigue, psychological suffering, itching intensity, facial esthetics, as well as changes in dental and smile esthetics. This scale is commonly graded from 0 to 10 and contains user instructions, allowing respondents to classify the outcome using numbers. In this system, 0 represents no pain and 10 represents maximal imaginable pain.

### *American Shoulder and Elbow Surgeons (ASES) Score*

The ASES score was developed to measure functional limitations and shoulder pain in people with musculoskeletal pathologies. Pain score was calculated from a single pain question on a visual analog scale (pain symptoms) and a function score from the sum of 10 questions addressing function using a 4‐point ordinal scale (physical function). Pain and function are weighted equally and the total score ranges from 0 to 100 points, where 0 is worst and 100 is best.

### *Constant–Murley Score (CMS)*

The CMS is a multi‐item functional scale assessing pain, activities of daily living, range of motion, and strength of the affected shoulder. Its score ranges from 0 to 100 points, representing worst and best shoulder function, respectively.

### *Simple Shoulder Test (SST)*

The SST measures functional limitations of the affected shoulder in people with shoulder dysfunction, and consists of 12 questions with dichotomous (1 = yes or 0 = no) response options. For each question, the patients indicated that they were able or were not able to do the activity (physical function). The scores range from 0 to 100, where 0 was worst and 100 was best, and are reported as the percentage of items that a person reports being able to do.

### *Statistical Analysis*

Descriptive statistics, including the mean and standard deviation (SD) for continuous variables, and the frequency and proportion of categorical variables were calculated. Statistical analysis was performed using SPSS for Windows (version 22; SPSS Inc., Chicago, Illinois, US). Comparisons between more than two groups were conducted using the Kruskal–Wallis test. A *P* value <0.05 was considered significant. The data are reported below.

## RESULTS

### *Patient Information*

The study cohort comprised 20 men and nine women, with an average age of 42.8 ± 13.5 years. A total of seven, six, and 16 patients had Rockwood type III, type IV, and type V separations, respectively. Seven patients were injured in traffic accidents and 22 by falling during sports.

### *Operative Details*

The mean duration of operation was 77.3 ± 14.1 min, and the mean blood loss was 67.1 ± 18.6 mL. The average postoperative follow‐up was 28 ± 10.2 months (Table 1).

### *Visual Analog Scale (VAS)*

The mean preoperative VAS score (baseline data) was 6.6 ± 1.3. At the 3‐month, 6‐month, and 12‐month follow‐up evaluation, the mean pain score, as measured from 1 to 10 on the VAS, was 4.2 ± 1.1, 2.7 ± 1.4, and 0.5 ± 0.7, respectively. The VAS score at 12 months postoperative was 6.1 higher than the preoperative VAS score (*P* < 0.01). No significant difference between the 6‐ and 12‐month follow‐up evaluations could be found (*P* > 0.05) (Table 2).

**TABLE 2:** Scores of the VAS for pain, Constant–Murley, SST, and ASES preoperatively and at 3, 6, and 12 months postoperatively (N = 29)

The Functional score	Preoperative	Postoperative 3rd month	Postoperative 6th month	Postoperative 12th month	*P* Value
VAS	6.6 ± 1.3	4.2 ± 1.1	2.7 ± 1.4	0.5 ± 0.7	<0.001
CS	30.3 ± 4.3	71.3 ± 6.4	88.2 ± 4.5	93.0 ± 2.4	<0.001
SST	2.3 ± 0.8	8.0 ± 1.6	10.9 ± 0.9	11.5 ± 0.6	<0.001
ASES	44.8 ± 3.1	77.3 ± 3.2	91.0 ± 2.6	92.1 ± 3.5	<0.001

ASES, American Shoulder and Elbow Surgeons; CS, Constant and Murley score; SST, simple shoulder test; VAS, visual analog scale.

### *American Shoulder and Elbow Surgeons(ASES) Score*

The mean preoperative, 3‐month, 6‐month, and 12‐month follow‐up evaluation ASES scores were 44.8 ± 3.1, 77.3 ± 3.2, 91.0 ± 2.6, and 92.1 ± 3.5 respectively. The ASES score at 12 months postoperative was 47.3 higher than the preoperative ASES score (*P* < 0.01). There was no significant difference in ASES score between the 6‐ and 12‐ month follow‐up evaluations (*P* > 0.05) (Table 2).

### *Constant–Murley Score (CMS)*

The mean preoperative, 3‐month, 6‐month, and 12‐month postoperative CMS were 30.3 ± 4.3, 71.3 ± 6.4, 88.2 ± 4.5, and 93.0 ± 2.4, respectively. The CMS at 12 months postoperative was 62.7 higher than the preoperative CMS (*P* < 0.01) between the baseline and 12‐month follow‐up data. No significant difference between the 6‐ and 12‐month follow‐up evaluations could be found (*P* > 0.05).

### *Simple Shoulder Test (SST)*

The mean preoperative SST score (baseline data) was 2.3 ± 0.8. At the 3‐month, 6‐month, and 12‐month follow‐up evaluation, the mean SST scores were 8.0 ± 1.6, 10.9 ± 0.9, and 11.5 ± 0.6, respectively. The SST score at 12 months postoperative was 9.2 higher than the preoperative SST score (*P* < 0.01). No significant difference between the 6‐ and 12‐month follow‐up evaluations could be found (*P* > 0.05) (Table 2).

### *Complications*

**Fig. 10:**
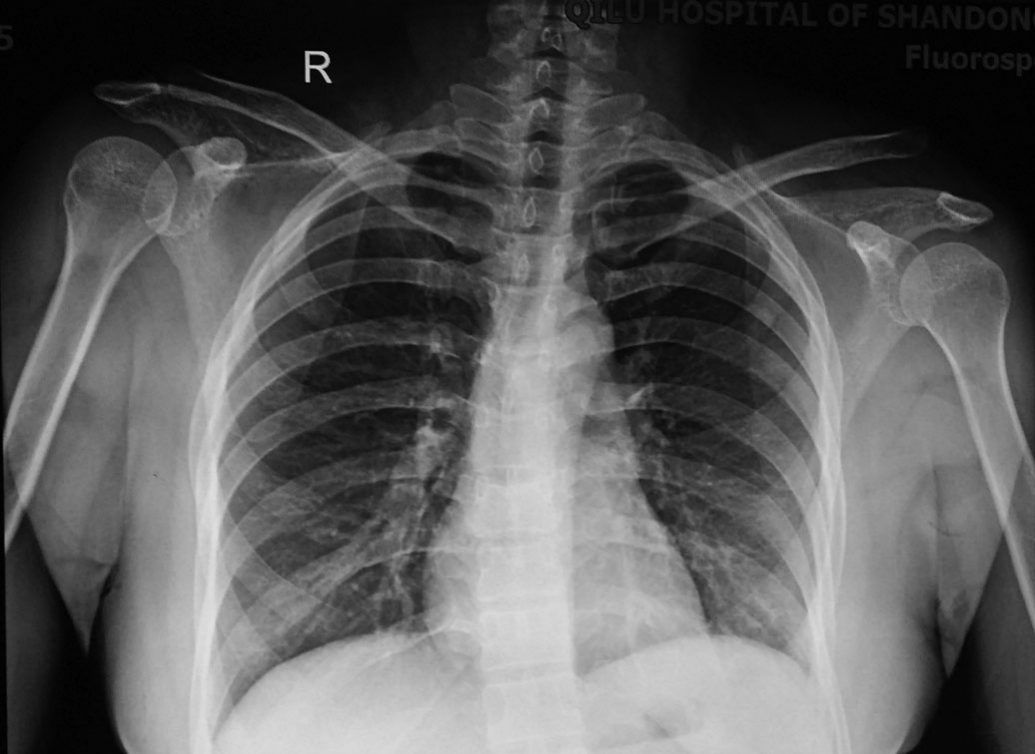
Female, 35 years old, left ACJ dislocation, type V.

**Fig. 11:**
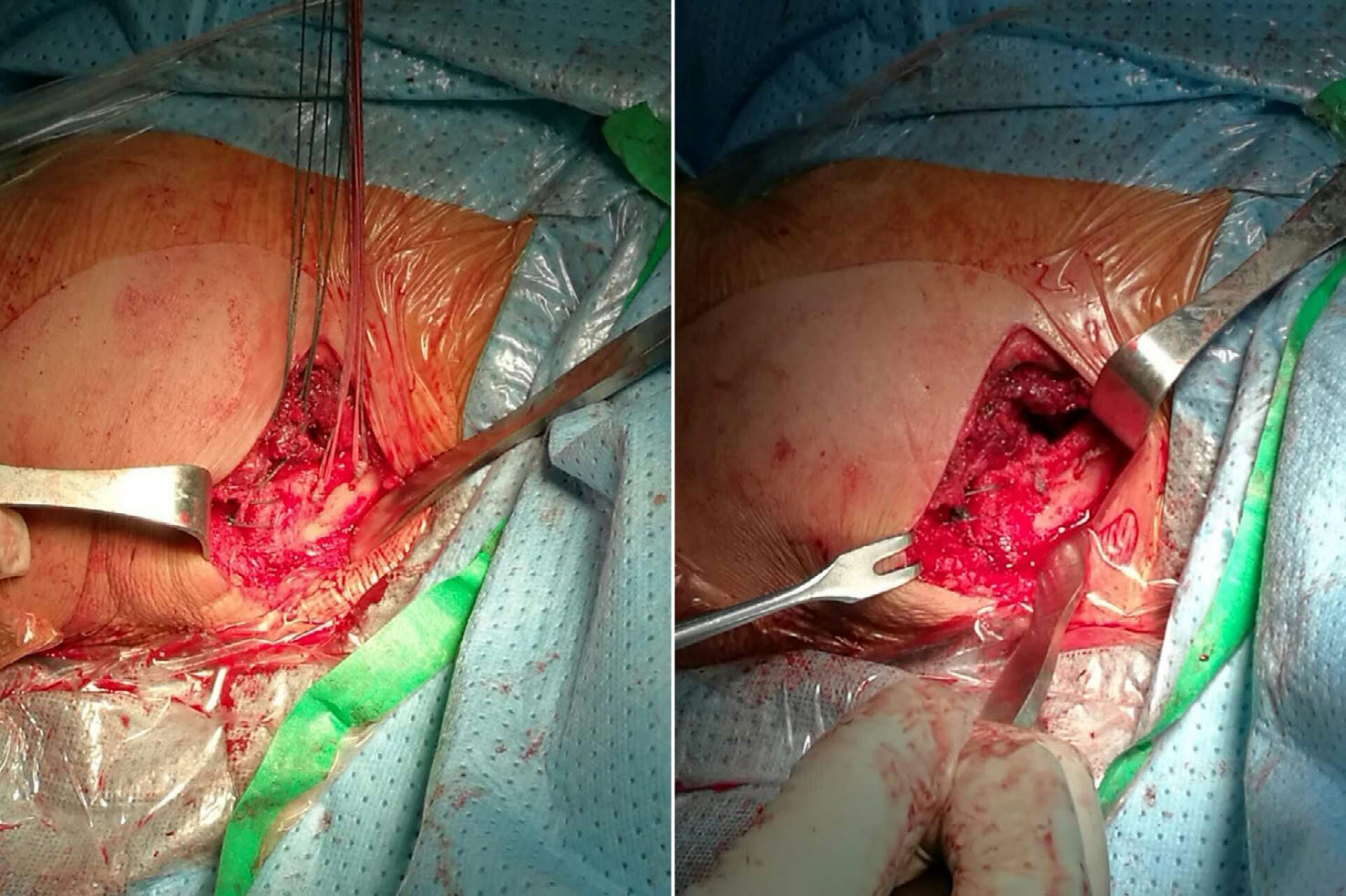
Intraoperative image before and after tightening the sutures.

**Fig. 12:**
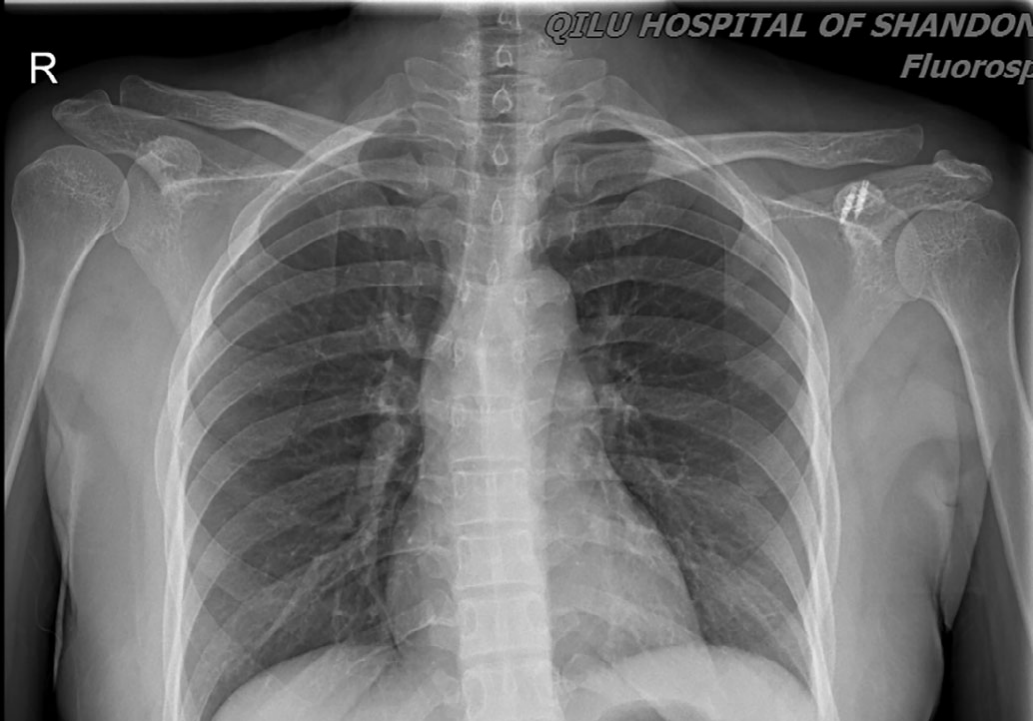
X‐Ray taken at 28‐month follow‐up visit.

**Fig. 13:**
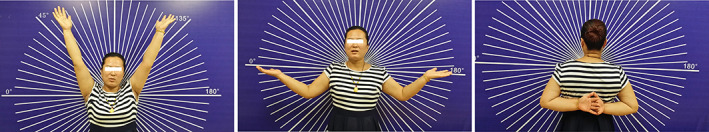
Patient felt no pain and had full range of motion and normal strength joint.

One patient developed a superficial wound infection 3 weeks postoperation. The wound healed after routine wound care. The patient presented good results at the time of the final follow‐up. No neurovascular complications were recorded (another typical case is shown in Figs 10, 11, 12, 13).

### *Radiographic Outcomes*

Anterior–posterior radiographs showed two partial loss of vertical reduction, which caused no adverse effects on patient clinical results. No horizontal instability was noted in all patients by axillary view and 3‐D‐CT.

## Discussion

### *Characteristics and Surgical Options of* *ACJ* *Dislocation*

No consensus has been reached regarding the treatment of high‐grade AC dislocation despite the prevalence of this injury^3^, ^5^, ^24^. The choice of an adequate surgical procedure is based on various factors, such as the surgeon's preference, the patient's activity level, and biomechanical properties of the surrounding ligaments^25^. More than 150 variations have been described to treat symptomatic ACJ separations^4^; however, the superiority of a single technique has not been defined up to this point. Regardless of the construct used, reduction must be maintained long enough for the biological healing process to occur^9^.

All surgical procedures for AC dislocation can be classified into four categories: (i) fixation of the AC and/or CC with hardware including screws and K‐wires; (ii) hook plates; (iii) fixation of the CC with suture buttons or anchors; (vi) reconstruction of CC ligaments with autograft or allograft tendon^26^. Temporary transarticular K‐wire fixation of the ACJ has led to unsatisfactory outcomes, including K‐wire breakage, migration, and loss of reduction^27^. Similarly, hardware failure and obligatory screw removal have decreased the popularity of CC cerclage or screw fixation^28^. The hook plate is a metal device that keeps the ACJ in a reduced position by hooking its tip under the acromion and fixing it to the clavicle with screws^29^, ^30^. Given the supra‐physiologic mechanical strength^31^ and good and excellent clinical outcomes^32^, ^33^, this technique is popular all over the world, especially in Europe. On the other hand, hook plates must be removed 8–12 weeks after surgery, a situation that involves the need of a second surgery. Common complications associated with hook plate fixation include functional limitations and pain^33^, clavicle fracture at the medial end of the plate^34^, subacromial impingement and rotator cuff tears^35^, upward cutting of the hook through the acromion^36^, acromial osteolysis^37^, and fracture^38^. Despite the timely plate removal, an increased risk of fracture of the distal clavicle after low‐energy trauma may also exist^39^. These implant‐related adverse effects may influence a patient's final functional outcome and hinder the clinical application of such procedures^40^.

### *Current Trends and Existing Problems*

Anatomic reconstruction of the CC and AC ligaments using tendon grafts and endobutton CC fixation in acute ACJ dislocation have rapidly gained popularity in the past few decades^8^, ^13^. Clavicle and/or coracoid fractures resulting from bone tunnels, which are usually 6 mm in diameter, are the main reasons that restrict these techniques. Several authors recommended the use of 3 mm bone tunnels to avoid the use of large bone tunnels to reduce either clavicle or coracoid fractures^41^, ^42^, ^43^.

### *Technical Characteristics and Key Technologies of this Study*

In the present study, the authors successfully maintained the ACJ in a reduced position using two suture anchors in patients with high‐grade AC dislocation. This technology offers the following important features: (i) Regardless of whether 3.5 (295 N) or 5.0 mm (331N) was used, two suture anchors provided comparable biomechanical strength compared with the native CC ligament complex of 589 N and had sufficient strength to withstand physiological loads and restore stability^12^, ^44^; (ii) Suture anchor implantation in the base of the coracoid process was easier and less dangerous for the neurovascular structures than passing a loop underneath the coracoid process^25^; (iii) Jerosch *et al*.^45^ evaluated eight different AC reconstruction techniques in a biomechanical study, observing the best restoration of anatomy with suture anchor fixation in the base of the coracoid process; (vi) When 3.5 mm suture anchors were used, both coracoid and clavicle tunnels were created by 2.0 mm drill bit or Kirschner wire, which needed smaller clavicle holes than those required for the tendon graft or endobutton procedure, thus minimizing the possibility of intraoperative and postoperative fractures; (v) Hardware removal was unnecessary, and implant‐related complication was avoided, indirectly reducing the cost for family members and the national healthcare system. Numerous studies have confirmed the feasibility and effectiveness of suture anchor fixation to repair ACJ separation^2^, ^21^, ^25^, ^46^, ^47^, ^48^. Nevertheless, all shoulders were immobilized in a sling for 6 weeks to ensure native CC ligament healing and prevent reduction loss.

Distal clavicle resection (Mumford procedure) may represent an effective solution to a painful old ACJ injury^5^, ^49^. Various modified Weaver–Dunn procedures which include distal clavicle excision have been widely used^21^, ^25^, ^50^. However, for acute AC injuries, a great controversy remains about whether distal clavicle resection should be conducted^3^, ^5^. Aliberti *et al*.^15^ reported that horizontal instability injuries are often neglected or poorly understood, resulting in difficult diagnosis, which may lead to high complication rates and failure after surgical stabilization. Consistent evidence indicates that stability of horizontal plane plays a decisive role over the clinical outcome^23^. Other studies considered that the remaining horizontal instability is the only factor that may lead to an adverse effect on final clinical outcome^51^. Several scholars explored stabilization methods in the horizontal plane to address this important issue^14^, ^52^, ^53^, ^54^. In this study, we exposed the ACJ, debrided and removed the damaged cartilage disc, reduced the dislocated joint under direct vision, and performed reliable fixation by two strands of heavy nonabsorbable sutures. On one hand, horizontal stability was obtained. On the other hand, this condition further augmented and protected the vertical stability of CC fixation.

### *Limitations*

This study encountered several limitations, including its retrospective analysis, small sample size, lack of comparative cohort, and the displacement of 2 mm considered as arbitrary quantification standard. Therefore, further studies involving randomized controlled trials with larger numbers of cases are needed.

### *Conclusion*

Currently, no single surgical technique has demonstrated superior results over other forms of fixation. The authors believe that the two‐suture anchor fixation method for CC ligament and suture augmentation for ACJ demonstrates a reliable alternative for the surgical treatment of acute AC dislocation. This technique restores the stable ACJ both vertically and horizontally and provides sufficient strength to hold the distal clavicle to the coracoid process for CC and AC ligament healing. Nevertheless, other factors require attention during the surgical procedure.

## References

[1] Mazzocca AD , Arciero RA , Bicos J . Evaluation and treatment of acromioclavicular joint injuries. Am J Sports Med, 2007, 35: 316–329. 17251175 10.1177/0363546506298022

[2] Mendes Júnior AF , Jd MN , Dias DM , LFd S , Loures EA , Labronici PJ . Functional and radiological outcomes of the surgical treatment of acute acromioclavicular dislocation with anchors associated with clavicle and scapula fixation. Rev Bras Ortop (Sao Paulo), 2019, 54: 649–656. 31875063 10.1055/s-0039-1697020 PMC6923650

[3] Frank RM , Cotter EJ , Leroux TS , Romeo AA . Acromioclavicular joint injuries: evidence‐based treatment. J Am Acad Orthop Surg, 2019, 27: e775–e788. 31008872 10.5435/JAAOS-D-17-00105

[4] Beitzel K , Cote MP , Apostolakos J , *et al* Current concepts in the treatment of acromioclavicular joint dislocations. Art Ther, 2013, 29: 387–397. 10.1016/j.arthro.2012.11.023 23369483

[5] Cook JB , Krul KP . Challenges in treating acromioclavicular separations: current concepts. J Am Acad Orthop Surg, 2018, 26: 669–677. 30138294 10.5435/JAAOS-D-16-00776

[6] Moatshe G , Kruckeberg BM , Chahla J , *et al* Acromioclavicular and coracoclavicular ligament reconstruction for acromioclavicular joint instability: a systematic review of clinical and radiographic outcomes. Arthroscopy, 2018, 34: 1979–1995.e1978. 29573931 10.1016/j.arthro.2018.01.016

[7] Boffano M , Mortera S , Wafa H , Piana R . The surgical treatment of acromioclavicular joint injuries. EFORT Open Rev, 2017, 2: 432–437. 29209519 10.1302/2058-5241.2.160085 PMC5702953

[8] Carofino BC , Mazzocca AD . The anatomic coracoclavicular ligament reconstruction: surgical technique and indications. J Shoulder Elbow Surg, 2010, 19: 37–46. 20188267 10.1016/j.jse.2010.01.004

[9] van Bergen CJA , van Bemmel AF , Alta TDW , van Noort A . New insights in the treatment of acromioclavicular separation. World J Orthop, 2017, 8: 861–873. 29312844 10.5312/wjo.v8.i12.861 PMC5745428

[10] Beitzel K , Obopilwe E , Chowaniec DM , *et al* Biomechanical comparison of arthroscopic repairs for acromioclavicular joint instability: suture button systems without biological augmentation. Am J Sports Med, 2011, 39: 2218–2225. 21841067 10.1177/0363546511416784

[11] Wylie JD , Johnson JD , DiVenere J , Mazzocca AD . Shoulder acromioclavicular and coracoclavicular ligament injuries: common problems and solutions. Clin Sports Med, 2018, 37: 197–207. 29525023 10.1016/j.csm.2017.12.002

[12] Walz L , Salzmann GM , Fabbro T , Eichhorn S , Imhoff AB . The anatomic reconstruction of acromioclavicular joint dislocations using 2 TightRope devices: a biomechanical study. Am J Sports Med, 2008, 36: 2398–2406. 18765674 10.1177/0363546508322524

[13] Venjakob AJ , Salzmann GM , Gabel F , *et al* Arthroscopically assisted 2‐bundle anatomic reduction of acute acromioclavicular joint separations: 58‐month findings. Am J Sports Med, 2013, 41: 615–621. 23371472 10.1177/0363546512473438

[14] Saier T , Venjakob AJ , Minzlaff P , *et al* Value of additional acromioclavicular cerclage for horizontal stability in complete acromioclavicular separation: a biomechanical study. Knee Surg Sports Traumatol Arthrosc, 2015, 23: 1498–1505. 24554242 10.1007/s00167-014-2895-7

[15] Aliberti GM , Kraeutler MJ , Trojan JD , Mulcahey MK . Horizontal instability of the acromioclavicular joint: a systematic review. Am J Sports Med, 2020, 48: 504–510. 31013137 10.1177/0363546519831013

[16] Williams G , Nguyen V , Rockwood C . Classification and radiographic analysis of acromioclavicular dislocations. Appl Radiol, 1989, 18: 29–34.

[17] Beitzel K , Mazzocca AD , Bak K , *et al* ISAKOS upper extremity committee consensus statement on the need for diversification of the Rockwood classification for acromioclavicular joint injuries. Art Ther, 2014, 30: 271–278. 10.1016/j.arthro.2013.11.005 24485119

[18] Mazzocca AD , Conway JE , Johnson S , *et al* The anatomic coracoclavicular ligament reconstruction. Operative Techniques in Sports Medicine, 2004, 12: 56–61.

[19] Salzmann GM , Paul J , Sandmann GH , Imhoff AB , Schöttle PB . The coracoidal insertion of the coracoclavicular ligaments: an anatomic study. Am J Sports Med, 2008, 36: 2392–2397. 18755935 10.1177/0363546508322887

[20] Rios CG , Arciero RA , Mazzocca AD . Anatomy of the clavicle and coracoid process for reconstruction of the coracoclavicular ligaments. Am J Sports Med, 2007, 35: 811–817. 17293463 10.1177/0363546506297536

[21] Jiang C , Wang M , Rong G . Proximally based conjoined tendon transfer for coracoclavicular reconstruction in the treatment of acromioclavicular dislocation. J Bone Joint Surg Am, 2007, 89: 2408–2412. 17974882 10.2106/JBJS.F.01586

[22] Tauber M , Koller H , Hitzl W , Resch H . Dynamic radiologic evaluation of horizontal instability in acute acromioclavicular joint dislocations. Am J Sports Med, 2010, 38: 1188–1195. 20360606 10.1177/0363546510361951

[23] Wellmann M , da Silva G , Lichtenberg S , Magosch P , Habermeyer P . [Instability pattern of acromioclavicular joint dislocations type Rockwood III: relevance of horizontal instability]. Der Orthopade, 2013, 42: 271–277. 23512005 10.1007/s00132-013-2085-1

[24] Chang N , Furey A , Kurdin A . Operative versus nonoperative management of acute high‐grade acromioclavicular dislocations: a systematic review and meta‐analysis. J Orthop Trauma, 2018, 32: 1–9. 29257778 10.1097/BOT.0000000000001004

[25] Shin SJ , Yun YH , Yoo JD . Coracoclavicular ligament reconstruction for acromioclavicular dislocation using 2 suture anchors and coracoacromial ligament transfer. Am J Sports Med, 2009, 37: 346–351. 19022989 10.1177/0363546508324968

[26] Lee S , Bedi A . Shoulder acromioclavicular joint reconstruction options and outcomes. Curr Rev Musculoskelet Med, 2016, 9: 368–377. 27645218 10.1007/s12178-016-9361-8 PMC5127941

[27] Modi CS , Beazley J , Zywiel MG , Lawrence TM , Veillette CJ . Controversies relating to the management of acromioclavicular joint dislocations. Bone Joint J, 2013, 95‐B: 1595–1602. 10.1302/0301-620X.95B12.31802 24293587

[28] Barnes CJ , Higgins LD , Major NM , Basamania CJ . Magnetic resonance imaging of the coracoclavicular ligaments: its role in defining pathoanatomy at the acromioclavicular joint. J Surg Orthop Adv, 2004, 13: 69–75. 15281402

[29] D B n E . Moethode zur operative Behandlung der akromioklavikularen Luxation. Chir Prax, 1976: 275.

[30] Wolter D , Eggers C . [Reposition and fixation of acromioclavicular luxation using a hooked plate]. Hefte Unfallheilkd, 1984, 170: 80–86. 6706651

[31] Vajapey SP , Bong MR , Peindl RD , Bosse MJ , Ly TV . Evaluation of the clavicle hook plate for treatment of acromioclavicular joint dislocation: a cadaveric study. J Orthop Trauma, 2020, 34: e20–e25. 31567796 10.1097/BOT.0000000000001632

[32] Jensen G , Katthagen JC , Alvarado LE , Lill H , Voigt C . Has the arthroscopically assisted reduction of acute AC joint separations with the double tight‐rope technique advantages over the clavicular hook plate fixation? Knee Surg Sports Traumatol Arthrosc, 2014, 22: 422–430. 23124627 10.1007/s00167-012-2270-5

[33] Kienast B , Thietje R , Queitsch C , Gille J , Schulz AP , Meiners J . Mid‐term results after operative treatment of Rockwood grade III‐V acromioclavicular joint dislocations with an AC‐hook‐plate. Eur J Med Res, 2011, 16: 52–56. 21463981 10.1186/2047-783X-16-2-52 PMC3353421

[34] Ding M , Ni J , Hu J , Song D . Rare complication of clavicular hook plate: clavicle fracture at the medial end of the plate. J Shoulder Elbow Surg, 2011, 20: e18–e20. 10.1016/j.jse.2011.06.005 21831666

[35] Hackenberger J , Schmidt J , Altmann T . [The effects of hook plates on the subacromial space–a clinical and MRT study]. Zeitschrift fur Orthopadie und ihre Grenzgebiete, 2004, 142: 603–610. 15472772 10.1055/s-2004-832323

[36] Gstettner C , Tauber M , Hitzl W , Resch H . Rockwood type III acromioclavicular dislocation: surgical versus conservative treatment. J Shoulder Elbow Surg, 2008, 17: 220–225. 18249565 10.1016/j.jse.2007.07.017

[37] Chiang CL , Yang SW , Tsai MY , Kuen‐Huang Chen C . Acromion osteolysis and fracture after hook plate fixation for acromioclavicular joint dislocation: a case report. J Shoulder Elbow Surg, 2010, 19: e13–e15. 10.1016/j.jse.2009.12.005 20303294

[38] Hoffler CE , Karas SG . Transacromial erosion of a locked subacromial hook plate: case report and review of literature. J Shoulder Elbow Surg, 2010, 19: e12–e15. 10.1016/j.jse.2009.10.019 20189416

[39] Nadarajah R , Mahaluxmivala J , Amin A , Goodier DW . Clavicular hook‐plate: complications of retaining the implant. Injury, 2005, 36: 681–683. 15826633 10.1016/j.injury.2004.08.010

[40] Lin HY , Wong PK , Ho WP , Chuang TY , Liao YS , Wong CC . Clavicular hook plate may induce subacromial shoulder impingement and rotator cuff lesion–dynamic sonographic evaluation. J Orthop Surg Res, 2014, 9: 6. 24502688 10.1186/1749-799X-9-6 PMC3922330

[41] Millett PJ , Horan MP , Warth RJ . Two‐year outcomes after primary anatomic coracoclavicular ligament reconstruction. Art Ther, 2015, 31: 1962–1973. 10.1016/j.arthro.2015.03.034 25998014

[42] Millett PJ , Warth RJ , Greenspoon JA , Horan MP . Arthroscopically assisted anatomic coracoclavicular ligament reconstruction technique using coracoclavicular fixation and soft‐tissue grafts. Arthrosc Tech, 2015, 4: e583–e587. 26900558 10.1016/j.eats.2015.06.007 PMC4722491

[43] Spiegl UJ , Smith SD , Euler SA , Dornan GJ , Millett PJ , Wijdicks CA . Biomechanical consequences of coracoclavicular reconstruction techniques on clavicle strength. Am J Sports Med, 2014, 42: 1724–1730. 24627576 10.1177/0363546514524159

[44] Lädermann A , Gueorguiev B , Stimec B , Fasel J , Rothstock S , Hoffmeyer P . Acromioclavicular joint reconstruction: a comparative biomechanical study of three techniques. J Shoulder Elbow Surg, 2013, 22: 171–178. 22541912 10.1016/j.jse.2012.01.020

[45] Jerosch J , Filler T , Peuker E , Greig M , Siewering U . Which stabilization technique corrects anatomy best in patients with AC‐separation? An Experimental Study. Knee Surg Sports Traumatol Arthrosc, 1999, 7: 365–372. 10639655 10.1007/s001670050182

[46] Breslow MJ , Jazrawi LM , Bernstein AD , Kummer FJ , Rokito AS . Treatment of acromioclavicular joint separation: suture or suture anchors? J Shoulder Elbow Surg, 2002, 11: 225–229. 12070493 10.1067/mse.2002.123904

[47] Fleischli JE . Editorial commentary: biomechanics of all suture anchors: what we know so far. Art Ther, 2018, 34: 2796–2798. 10.1016/j.arthro.2018.07.010 30286879

[48] Zhang JW , Li M , He XF , Yu YH , Zhu LM . Operative treatment of acromioclavicular joint dislocation: a new technique with suture anchors. Chin J Traumatol, 2014, 17: 187–192. 25098843

[49] Snyder SJ , Banas MP , Karzel RP . The arthroscopic Mumford procedure: an analysis of results. Art Ther, 1995, 11: 157–164. 10.1016/0749-8063(95)90061-6 7794427

[50] Boileau P , Old J , Gastaud O , Brassart N , Roussanne Y . All‐arthroscopic Weaver‐Dunn‐Chuinard procedure with double‐button fixation for chronic acromioclavicular joint dislocation. Art Ther, 2010, 26: 149–160. 10.1016/j.arthro.2009.08.008 20141978

[51] Scheibel M , Dröschel S , Gerhardt C , Kraus N . Arthroscopically assisted stabilization of acute high‐grade acromioclavicular joint separations. Am J Sports Med, 2011, 39: 1507–1516. 21436458 10.1177/0363546511399379

[52] Cisneros LN , Sarasquete Reiriz J , Besalduch M , *et al* Horizontal and vertical stabilization of acute unstable acromioclavicular joint injuries arthroscopy‐assisted. Arthrosc Tech, 2015, 4: e721–e729. 26870653 10.1016/j.eats.2015.07.014 PMC4738758

[53] Theopold J , Schöbel T , Fischer JP , *et al* Acromioclavicular joint reconstruction: an additional acromioclavicular cerclage does not improve horizontal stability in double coraco‐clavicular tunnel technique. Knee Surg Sports Traumatol Arthrosc, 2019, 27: 3827–3834. 31420689 10.1007/s00167-019-05674-1

[54] Jordan RW , Malik S , Bentick K , Saithna A . Acromioclavicular joint augmentation at the time of coracoclavicular ligament reconstruction fails to improve functional outcomes despite significantly improved horizontal stability. Knee Surg Sports Traumatol Arthrosc, 2019, 27: 3747–3763. 30267185 10.1007/s00167-018-5152-7

